# Molecular In-Depth on the Epidemiological Expansion of SARS-CoV-2 XBB.1.5

**DOI:** 10.3390/microorganisms11040912

**Published:** 2023-03-31

**Authors:** Fabio Scarpa, Ilenia Azzena, Chiara Locci, Marco Casu, Pier Luigi Fiori, Alessandra Ciccozzi, Silvia Angeletti, Elena Imperia, Marta Giovanetti, Antonello Maruotti, Alessandra Borsetti, Roberto Cauda, Antonio Cassone, Allegra Via, Stefano Pascarella, Daria Sanna, Massimo Ciccozzi

**Affiliations:** 1 Department of Biomedical Sciences, University of Sassari, 07100 Sassari, Italy; 2 Department of Veterinary Medicine, University of Sassari, 07100 Sassari, Italy; 3 Azienza Ospedaliera Universitaria (AOU) Sassari, 07100 Sassari, Italy; 4 Unit of Medical Statistics and Molecular Epidemiology, University Campus Bio-Medico of Rome, 00128 Rome, Italy; 5 Unit of Clinical Laboratory Science, Department of Medicine and Surgery, University Campus Bio-Medico of Rome, 00128 Rome, Italy; 6 Research Unit of Laboratory, University Hospital Campus Bio-Medico, 00128 Rome, Italy; 7 Unit of Gastroenterology, Department of Medicine, University Campus Bio-Medico of Rome, 00128 Rome, Italy; 8 Instituto Rene Rachou, Fundação Oswaldo Cruz, Belo Horizonte 30190-009, Minas Gerais, Brazil; 9 Science and Technology for Sustainable Development and One Health, University of Campus Bio-Medico of Rome, 00128 Rome, Italy; 10 Department GEPLI, Libera Università Ss Maria Assunta, 00193 Rome, Italy; 11 National HIV/AIDS Research Center, Istituto Superiore di Sanità, 00161 Rome, Italy; 12 UOC Malattie Infettive, Infectious Disease Department, Fondazione Policlinico Universitario Agostino Gemelli IRCCS, 00168 Rome, Italy; 13 Center of Genomic, Genetic and Biology, 53100 Siena, Italy; 14 Department of Biochemical Sciences “A. Rossi Fanelli”, Sapienza Università di Roma, 00185 Rome, Italy

**Keywords:** COVID-18, SARS-CoV-2, molecular epidemiology, pandemics, phylogenomics, XBB.1.5

## Abstract

Since the beginning of the pandemic, the generation of new variants periodically recurs. The XBB.1.5 SARS-CoV-2 variant is one of the most recent. This research was aimed at verifying the potential hazard of this new subvariant. To achieve this objective, we performed a genome-based integrative approach, integrating results from genetic variability/phylodynamics with structural and immunoinformatic analyses to obtain as comprehensive a viewpoint as possible. The Bayesian Skyline Plot (BSP) shows that the viral population size reached the plateau phase on 24 November 2022, and the number of lineages peaked at the same time. The evolutionary rate is relatively low, amounting to 6.9 × 10^−4^ subs/sites/years. The NTD domain is identical for XBB.1 and XBB.1.5 whereas their RBDs only differ for the mutations at position 486, where the Phe (in the original Wuhan) is replaced by a Ser in XBB and XBB.1, and by a Pro in XBB.1.5. The variant XBB.1.5 seems to spread more slowly than sub-variants that have caused concerns in 2022. The multidisciplinary molecular in-depth analyses on XBB.1.5 performed here does not provide evidence for a particularly high risk of viral expansion. Results indicate that XBB.1.5 does not possess features to become a new, global, public health threat. As of now, in its current molecular make-up, XBB.1.5 does not represent the most dangerous variant.

## 1. Introduction

As of 26 February 2023, the total number of confirmed cases of COVID-19 globally reported has amounted to over 758 million with over 6.8 million deaths [1]. Since its first identification as a novel coronavirus, which caused an outbreak in Wuhan (China) in December 2019 [2,3], many SARS-CoV-2 waves have occurred (see e.g., Meyer et al. [4]) due to the generation of new variants and/or sublineages [5]. Indeed, the continuing and uninterrupted evolution of SARS-CoV-2 has caused variation at a nucleotide level that inevitably translates as the generation of new variants [5], and infections are likely to remain a problem for the time being in most countries.

SARS-CoV-2 is a positive-sense single-stranded RNA virus and, because of the occurrence of errors during the process of RNA replication [6], the rate of nucleotide substitution is fast, with a high error rate and quickness that affect the transmissibility of the virus [7]. Owing to this rapid evolution, mainly shaped by natural selection, the generation of new variants periodically recurs [5,8].

The World Health Organization (WHO) has labeled the variants of concern (VoC) as Alpha, Beta, Gamma, Delta and Omicron [9], to which all their descendant lineages should be added.

Currently, the Omicron VoCs and their descendant lineages remain the dominant variants circulating globally, with prevalence rates that vary in accordance with the expansion capabilities of the sublineage [10]. The SARS-CoV-2 variant BA.5 became worldwide dominant in early 2022 and, as such, persists with one of its sublineages. Indeed, the most recent direct descendant of BA.5 is the variant BQ.1, which is nicknamed Cerberus [10], and represents the most common lineage spread worldwide with a prevalence rate of 44% as of 30 January 2023 [11]. However, the occurrence of recombination events must not be overlooked, which, together with sequence re-assortment, represent the main contributor to RNA virus evolution [12]. The most recent SARS-CoV-2 recombinant is the lineage labeled as XBB (nicknamed Gryphon [13]). It has been generated by the recombination between BJ.1 (as Donor) and BM.1.1.1 (as Acceptor), both belonging to the BA.2 lineage [13,14,15,16]. As always happens at the beginning of the evolutionary path of a new generated lineage, many concerns arose initially, mainly due to its supposed strongly immunoevasive properties [17]. However, after a genome-based survey aimed to verify its expansion capabilities and to perform a comparison between XBB and its parental lineages, Scarpa et al. [13] proved that the so-called gryphon variant, although being strongly immunoevasive, does not present evidence of particularly dangerous or high expansion capability. Likewise, similar concerns are now directed to its descendant, the XBB.1.5 sublineage, which is nicknamed Kraken [18,19]. Sublineage XBB.1.5 belongs to the GSAID Clade 23A [11] and, from 22 October 2022 to 21 February 2023, a total of 45.193 Omicron XBB.1.5 variant sequences were reported from 74 countries [20] with a genome worldwide prevalence of about 21% [11]. However, it should be pointed out that most of these sequences are from the United States of America (72.2%) and the United Kingdom (7.3%), while, as of late February 2023, other countries show a genome prevalence of no more than 20% [20].

The lineage XBB.1.5 presents in the spike protein sequence the same point mutations of interest (see Table S1) as its progenitor XBB (i.e., K417N, S477N, N501Y and P681H) [21], except for mutation at position 486, which in XBB.1.5 consists of F486P [22] while in XBB the phenylaniline (F) is replaced by a serine (S) [21]. Regarding the genetic characteristics of XBB.1.5, the new lineage is expected to contribute to an increase in the number of confirmed cases globally. Indeed, although there is currently no indication of increased severity, there is moderate evidence suggesting an increased risk of transmission as well as evasion of the immune system. The overall concern with XBB.1.5 is the F486P substitution, which is quite rare when comparing all other lineages with the wild type Wuhan-1 [23]. This mutation plays an, as yet, unknown role, although it cannot be ruled out that, like many new mutations, it may confer the virus the ability to escape the immune system better than other lineages. For this reason, the WHO recommends prioritizing studies aimed at better understanding this lineage. In such a context, here, we followed a genome-based integrative approach where results from genetic variability/phylodynamics analyses were compared and integrated with structural and immunoinformatic studies to obtain as comprehensive a viewpoint as possible. The research is aimed at providing a molecular in-depth analyses on the epidemiological expansion of SARS-CoV-2 XBB.1.5 in order to perform an uninterrupted monitoring (see e.g., Scarpa et al. [7,10,13,14,15]).

## 2. Materials and Methods

### 2.1. Phylodynamics Analyses

The first genomic epidemiology of SARS-CoV-2 XBB.1.5 Omicron subvariant was reconstructed using nextstrain/ncov [24] on a dataset (n = 1174) including all complete genomes belonging to the GSAID Clade Omicron [11]. Genomes were filtered for relevance (avoiding the inclusion of genomes with less than 0.01% differences) and for high quality and coverage. See File S1 for details on the used genomes and authorship.

After the first genomic assessment, a further dataset composed by all available genomes belonging to the SARS-CoV-2 XBB.1.5 lineage (n = 216) was further analyzed. For both datasets, genomes were aligned using the algorithm L-INS-I implemented in Mafft 7.471 [25], producing a dataset of whole genomes 29,720 bp long. Manual editing was performed using the Unipro UGENE v.35 software [26]. jModeltest 2.1.1 [27] was used to find the best performing probabilistic model of genome evolution with a maximum likelihood optimized search. Phylogenomic relationships among variants and time of divergence were investigated applying Bayesian Inference (BI) with BEAST 1.10.4 [28] using runs of 200 million generations under several demographic and clock models. To infer the best representative output, the selection of the best performing model for dating inferences was achieved by testing both strict and uncorrelated log-normal relaxed clock models. Both clock models were further tested under both parametric demographic (constant population size, exponential population growth and expansion population growth) and piecewise-constant (Bayesian Skyline) models. Selection was performed by means of the Bayes Factor test [29], comparing the 2lnBF of the marginal likelihoods values as described in Mugosa et al. [30]. For this screening, only values of ESS (effective sample size) ≥ 200 were retained. The maximum clade credibility tree was drawn and annotated by means of the TreeAnnotator software from the BEAST package. Phylogenetic trees were edited and visualized using FigTree 1.4.0 [31]. The BEAST software was also used to co-estimate the evolutionary rate, Bayesian Skyline Plot (BSP) and lineages through times on a subset composed of 216 whole genomes of XBB.1.5 (with high quality and high coverage sublineage), by means of runs of 300 million generations under the Bayesian Skyline Model with the uncorrelated log-normal relaxed clock model. The occurrence of a recombination event within the whole dataset [13] made the molecular dating non-applicable because of the noisy in the temporal signal (see e.g., Scarpa et al. [32]). Dating was applied only on the subset of XBB.1.5. All datasets were built using genomes downloaded on 13 January 2023 from the GSAID portal [33]. Genomes included in the datasets were filtered for high quality and coverage.

### 2.2. Structural and Molecular Dynamics Analyses

Homology models of the variant Spike RBDs and NTDs were built using Modeller 10.3 [34]. Model structures were displayed and analyzed with the graphic program PyMOL [35]. Foldx 5.0 was applied to optimize the side chain conformation of the obtained models using the function “RepairPDB” [36]. To sample the fluctuations of the side chain conformations and interactions, 100 homology models of the RBD and NTD domains, and the RBD–ACE2 complex were determined with Modeler. Indeed, the Modeler refinement stage of the homology modelling produces alternative models differing for conformational details, among which are side chain rotamers. Each model was optimized using the Foldx 5.0 “RepairPDB” function. Structural properties were calculated for all models to evaluate their average and standard error. Net charges were predicted using PROPKA3 [37], setting pH = 7.0 as the reference pH, though not necessarily reflecting the physiological environment. Surface electrostatic potential was calculated with the program APBS [38] and displayed as a two-dimensional projection with the SURFMAP software [39]. SURFMAP implements a method of “molecular cartography”, by means of which a protein’s three-dimensional surface can be projected onto a two-dimensional plane. In this way, the distribution of different physicochemical features over the protein surface can be analyzed and compared. Interaction energy between the Spike RBD and ACE2 were predicted with tools based on different approaches: “AnalyseComplex” of the Foldx 5.0 suite, PRODIGY [40] and MM/GBSA (Molecular Mechanics/Generalized Born Surface Area) HawkDock [41]. Foldx 5.0 uses an empirical force field that describes the different free energy terms including, among others, electrostatic interactions, hydrogen bonds, desolvation and van der Waals contacts. PRODIGY estimates the binding affinity between two protein interfaces based on the number and type of interface residue pairwise contacts. MM/GBSA HawkDock calculates binding free energies for macromolecules by combining molecular mechanics calculations and continuum solvation methods. In silico mutagenesis was obtained with the built-in functions available within PyMOL. In silico alanine scanning of the residues at the interface between RBD and ACE2 was carried out using the method available via the web server DrugScorePPI [42]. The method is a fast and accurate computational approach to predict changes in the binding free energy when each residue at the subunit interface is in silico mutated into alanine. Prediction of epitopes recognized by B lymphocytes and of peptides potentially binding the MHC-I receptors was carried out with the programs BepiPred 3.0 [43] and netMHCpan 4.1 [44], respectively. Prediction of the MHC-I binding peptides was carried out with the program netMHCpan using a 9-residue peptide probe and HLA-A*02:01 as the test allele.

Molecular dynamics was applied to interpret the behavior of the P486 residue in XBB.1.5 that replaces S486 in XBB.1 by comparing the trajectories calculated for RBD in the two cases. Molecular dynamics was carried out with the program GROMACS 2020.1 [45] using the force field AMBER99SB-ILDN [46]. The RBD structure was solvated in a truncated octahedron box with TIP3P water molecules and a 2.0 nm distance from the system to the box edge. The solvated system was neutralized and set to a concentration of 0.15 M NaCl. All the simulations were calculated in periodic boundary conditions. The system was minimized with the steepest descent minimizer until convergence, namely, until no change in energy between successive steps was detected. After minimization, the system was subjected to 100 ps of NVT and 100 ps of NPT equilibration at 300 K with a modified Berendsen thermostat (time constant 1 ps). LINCS algorithm was applied to constrain the bond lengths. Electrostatic forces were calculated with the Particle Mesh Ewald method [47] using a grid spacing of 0.16 nm. A cutoff of 1.0 nm was set for short-range electrostatic and van der Waals interactions. The production simulation was run for 100 ns with a 2 fs time-step. Trajectories were visualized with the VMD 1.9.3 [48] graphic program and analyzed with the GROMACS tools and XMGRACE software package [49].

## 3. Results

**Figure 1:**
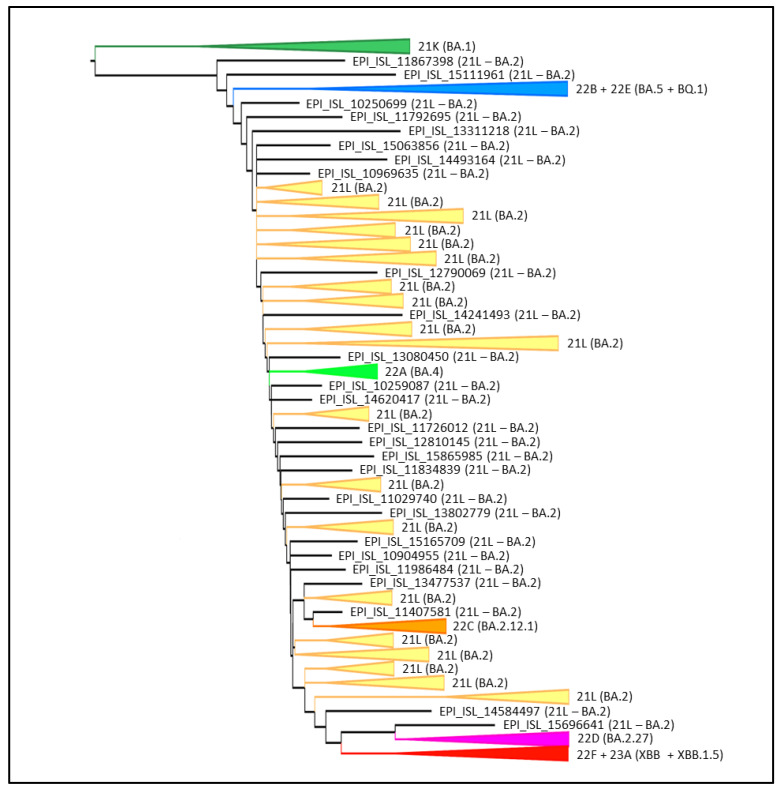
Phylogenomic reconstruction. Highlight of the Omicron clade in the phylogenetic tree of a representative global subsample of 1174 SARS-CoV-2 genomes sampled between January 2022 and February 2023. The tree was edited and visualized using FigTree 1.4.0 [31]. The generated figure was edited using the software GIMP 2.8 (available at https://www.gimp.org/downloads/oldstable/, accessed on 20 February 2023).

Phylogenomic reconstruction (Figure 1) indicates that all XBB.1.5 genomes clustered together forming the GSAID Clade 23A, which, in turn, clusters within its progenitor clade XBB and other sublineages constituting the GSAID Clade 22F. The group composed by Clade 22F + Clade 23A shows a sister–clade relationship with Clade 22D (i.e., BA.2.75 variant) + XBH (21L).

**Figure 2:**
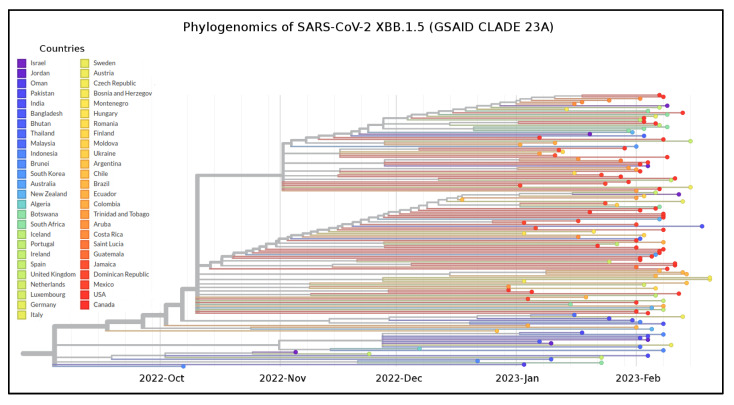
Phylogenomic time-scaled reconstruction of XBB.1.5. Highlight of the GSAID Clade 23 A in a time-scaled phylogenetic tree of a representative global subsample of 141 SARS-CoV-2 XBB.1.5 genomes sampled between October 2022 and February 2023, labeled according to countries of origin. The tree was edited and visualized using FigTree 1.4.0 [31]. The generated figure was edited using the software GIMP 2.8 (available at https://www.gimp.org/downloads/oldstable/, accessed on 20 February 2023).

Among the sampled lineages, the only variants that constitute a monophyletic group are XBB.1.5, BQ.1, BA.1, BA.4, BA.2.12.1 and BA.2.75, while other lineages constitute paraphyletic and polyphyletic groups. The temporal origin reconstruction placed the common ancestor to XBB.1.5 around 10 October 2022 (date confidence interval: 7 September 2022–30 October 2022) (Figure 2). The XBB.1.5 clade showed the lack of geographic structure both at the countries (Figure 2) and WHO regions levels (Figure S1).

**Figure 3:**
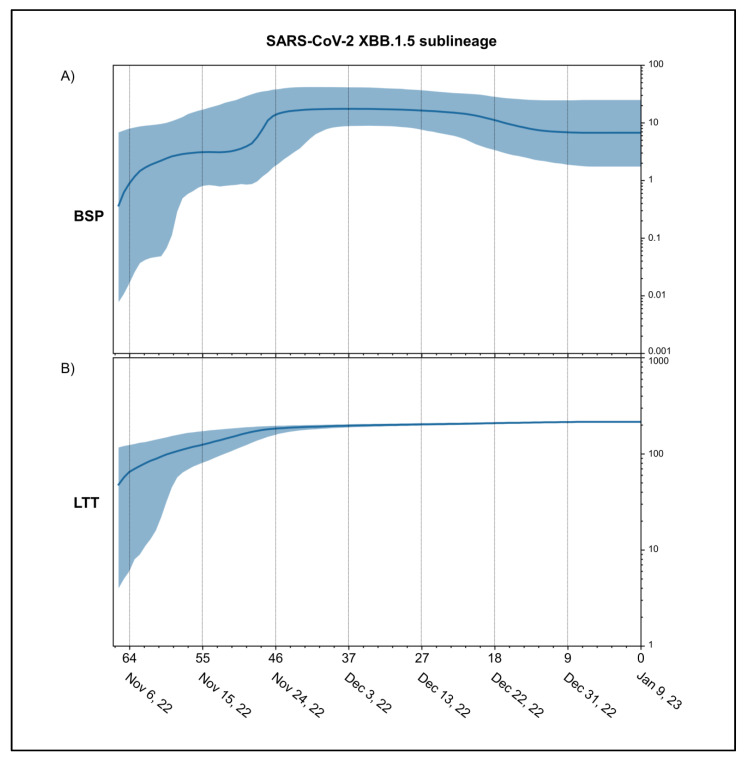
Demographic expansion of SARS-CoV-2 XBB.1.5. Bayesian Skyline Plot (**A**) and lineages through times (**B**) of SARS-CoV-2 XBB.1.5 variant. The viral effective population size (*y*-axis) is shown as a function of days (*x*-axis). The solid area represents the 95% high posterior density (HPD) region.

Results of the Bayes Factor on all datasets revealed that the Bayesian Skyline Model under the log-normal uncorrelated relaxed clock model fitted data significantly better than the other tested demographic and clock models with a value of 2lnBF = 26.4. Bayesian Skyline Plot (BSP) (Figure 3A) shows that the viral population size peaks, after several fluctuations, about 46 days before 9 January 2023 (i.e., 24 November 2022). Thereafter, a plateau phase begins with a slight reduction in population size, hence the genetic variability, about 16 days before 9 January 2023 (i.e., 24 December 2022). The lineages through the time plot (Figure 3B) indicates that the increase in the number of lineages stopped about 46 days before 9 January 2023 (i.e., 24 November 2022).

The evolutionary rate co-estimated with BSP and lineages through times amounts to 6.9 × 10^−4^ [95% HPD 5.3 × 10^−4^–8.5 × 10^−4^] subs/sites/years.

The recombinant XBB and its descendant variants XBB.1 and XBB.1.5 were compared. Mutated sites of XBB, XBB.1 and XBB.1.5 are shown in Table S1. The NTD domain is identical for XBB.1 and XBB.1.5, whereas their RBDs only differ for the mutations at position 486, where the original Wuhan Phe (conserved in BA.2) is replaced by a Ser in XBB and XBB.1, and a Pro in XBB.1.5. The predicted net charges of the RBD and NTD domains for the four variants are reported in Table 1.

**Figure 4:**
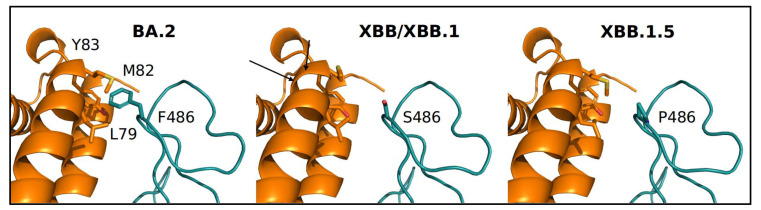
Comparison of the portion of RBD–ACE2 interface at position 486 in the four variants. RBD and ACE2 are represented as deep teal and orange ribbons, respectively. Relevant side chains are displayed as labeled stick models.

Interestingly, the NTD of the XBB descendant of BA.2 displays a negative net charge caused mainly by the introduction of two Glu residues at sequence positions 183 and 216 (Figure S1). On the contrary, the RBD displays a similar positive charge comparable with that observed in the first Omicron variant [13]. The interaction energy of the ACE2–RBD complex (Figure 4) for the variants at hand were calculated with three alternative methods relying on different criteria.

Results are reported in Table 2. All three methods suggest, within the limits of their accuracy and models, that the RBD of BA.2 tends to have a more stable interaction with ACE2 than all the XBB variants.

However, two out of three methods assign to XBB1.5 an interaction energy tendentially more stable than the cognate XBB variants. The energy differences should be considered with caution in consideration of the magnitude of the standard error. For a more thorough interpretation of the interaction energy differences, an assessment of the contribution of the single residues to the interface stability was carried out using the MM/GBSA method that, besides calculating the overall interaction energy, can assign to each interface residue its Gibbs energy contribution to the interaction. The focus was on site 486 of RBD, where XBB.1 and XBB.1.5 present different residues (Table 3).

Results suggest that the substitution of the original F486 with Ser in XBB and XBB.1 destabilizes the interface, whilst the introduction of Pro observed in XBB.1.5 seems to partially restore the free energy contribution to the interface stability. It should be considered that the DrugScorePPI results indicate F486 not to be a major hotspot of the interface, as also found in Scarpa et al. [13].

BepiPred analysis indicates that the predicted B-cell epitopes distribution is very similar among the four variants except for the region around the NTD sequence encompassed by positions 76 and 85 corresponding to the BA.2 peptide 76-TKRFDNPVLP-85. BepiPred predicts a B-epitope in XBB, XBB.1 and XBB1.5, where A83 replaces V83 and not in the BA.2 peptide. Of note, the highest scoring peptides (defined by the algorithm as “Strong binders”) are identical in all four variants. One difference was found instead among the “Weak binder” peptides. Indeed, NetMHCpan predicts peptide 364-VLYNFAPFF-372 to be a “Weak binder” only in BA.2 RBD. Interestingly, BA.2 L365 is replaced by Ile in the corresponding XBB, XBB.1 and XBB.1.5 peptides.

**Figure 5:**
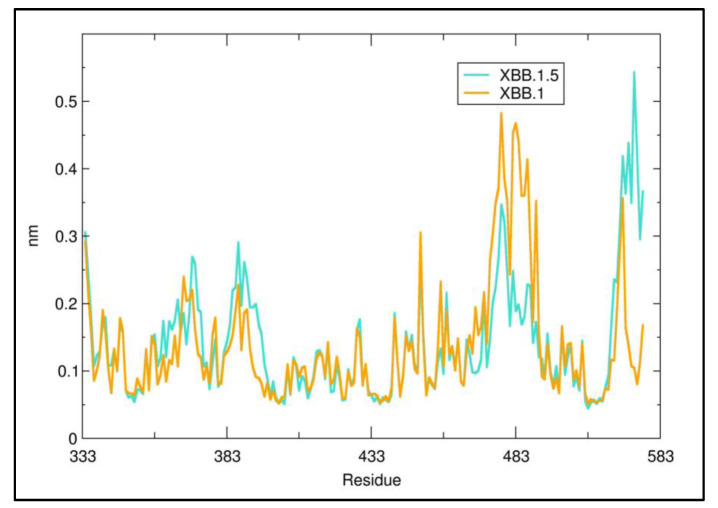
Residue RMSF of XBB.1 and XXB.1.5. The red arrow marks the approximate sequence position 486.

Molecular dynamics was applied to interpret the effect of P486 in XXB.1.5 versus Ser in XBB.1. The RMSF (Root Mean Square Fluctuation) plot calculated for the entire 100 ns simulation at the residue level (Figure 5) suggests that the presence of P486 decreases the local flexibility of the mainchain with respect to S486 in XBB.1. This may provide a rational basis to explain the relatively higher predicted stability of the complex RBD–ACE2 for XBB1.5 (Table 2).

## 4. Discussion

The XBB.1.5 lineage is one of the most recent emerging SARS-CoV-2 variants and represents the first descendant of the recombinant XBB for which doubts on public health arose. Indeed, XBB.1.5 is considered by the FDA (Food and Drug Administration) as one of the variants that carry one or more mutations that might potentially affect some vaccines or treatments [50]; for this reason, EvuSheld is not currently authorized for emergency use in the U.S. [50]. Nevertheless, it should be pointed that the new mutation carried by XBB.1.5 plays a yet unknown role. As XBB.1.5, like all newly emerging variants, requires attention, a thorough study of its ability to expand and increase viral contagiousness was deemed to be necessary.

Thus, we performed an in-depth genomic analysis to identify the evolutionary patterns of the SARS-CoV-2 XBB.1.5 lineage across all genomes available in GSAID as of 13 January 2023. We also carried out a structural analysis of XBB.1.5 point mutations at the interface of the RDB–ACE2 complex.

Phylogenomic reconstruction indicates that the genomes of XBB.1.5 (GSAID Clade 23A) all cluster together with the genomes of XBB and its descendants in the GSAID Clade 22F. XBB.1.5 showed a sister–clade relationship with BA.2.75 (GSAID Clade 22D). This is not surprising, as the parental lineage that generated XBB belongs to the Pango lineage BA.2 and BM.1.1.1, the acceptor (sensu Focosi and Maggi [51]) of the recombination event, is a direct descendant of BA.2.75 [13]. The evolutionary pattern of XBB.1.5 suggested by the phylogenomic reconstruction depicts an epidemic scenario very similar to those of several previous variants such as BA.2.75 (GSAID Clade 22D), BA.2.12.1 (GSAID Clade 22C) [14] and BQ.1 (GSAID Clade 22E) [10] that appeared as evolutionary blind backgrounds. Interestingly, this condition has also been identified for the original recombinant XBB and its first descendant XBB.1, which are now known not to exhibit characteristics of epidemiologically dangerous lineages. Similarly, XBB.1.5 shows a length of branches that suggests a lack of that rapid diversification, which, in fact, is the typical characteristic of a highly expansive, dangerous lineage early in its evolutionary path.

The common ancestor of all available genomes of XBB.1.5 is temporally placed around 10 October 2022. This dating is fully consistent with the first detected genome of XBB.1.5 (for which a complete sampling date is available), which appeared in India on 8 November 2022. Unlike its progenitor, which spread mainly in South Asia, Malaysia and Singapore [13], XBB.1.5 appeared more common in the region of the Americas from the beginning of its spread. Currently, it has been detected in 74 countries [20]. Its initial speed of diffusion suggested to the WHO the need to revise the confidence level of the risk assessment from low on 11 January 2023 to moderate on 25 January 2022 [52]. However, the Bayesian Skyline Plot (BSP) reconstruction shown here, estimated on high quality whole genomes of XBB.1.5 (sampling collection range: 8 November 2022–11 January 2023), indicates low levels of genetic variability with very few fluctuation points over time. Indeed, after the moderate increase of genetic variation, around 28 November 2022, the viral genetic variability and population size reached the peak and the plateau phase began. Then, shortly after (on 28 December 2022), a slight reduction in population size and, thus, in genetic variability occurred. The reconstruction of lineages through time indicates that the increase in the number of lineages reached the peak at the same time as the viral population size peaked. This is quite unusual; indeed, typically, the highest number of lineages appears at least a few weeks before the increase in the genetic variability. During the plateau phase, genetic variability (and, consequently, viral population size) showed little fluctuation with a slight reduction about 20 days before 11 January 2023 (i.e., 22 December 2022). Currently, the viral population size appears to be stable and flattened. It is important to note that this is not the typical trend of a lineage that is about to grow rapidly in terms of population size, expansion and growth in the number of lineages, as was seen at the beginning of the pandemic, when diversity increased rapidly according to a steep curve (e.g., Lai et al. [53]). In contrast, the observed trend of XBB.1.5 is similar to what was observed with several recent SARS-CoV-2 variants such as BA.2.75, BQ.1 and XBB, which, akin to XBB.1.5, initially caused concern, but after a comprehensive genome-based study [10,13,14], they were found to show no evidence of rapid, global spreading. Some of them (XBB and BA.2.75) spread even slower than other variants [13,14]. Overall, XBB.1.5, as BA.2.75, BQ.1 and XBB before, presents a typical scenario of an evolutionary lineage with new mutations in comparison to its direct ancestor but no advantage (at least at present) that would lead to an abnormal growth. Furthermore, the maximum number of lineages was reached in the middle of November 2022 and has remained stationary until now. This further supports the absence of an increase in the number of new haplotypes. The estimated evolutionary rate for XBB.1.5 (6.9 × 10^−4^ subs/site/year) further accounts for the low genetic variability and limited capacity for strong demographic expansion. The evolutionary rate of the variant BA.5, which remained dominant worldwide for several months in 2022, was slightly higher (7.4 × 10^−4^ subs/site/year) [14]. It should be noted that BA.5 had been circulating for several months before peaking, and its plateau phase presented several fluctuations of the viral population size [14]. It should be recalled here that, at the start of the current pandemic, the evolutionary rate of SARS-CoV-2 Wuhan-Hu-1 strain was around 6.58 × 10^−3^ subs/site/year [54], i.e., roughly ten times faster than XBB.1.5.

Furthermore, XBB.1.5, as all these recent BA.2 and BA.5-derived Omicron sub-variants, has remained so far confined to selected regional areas of the USA [55], despite its high transmissibility and immune-evasion potential, as reported by “COVID data tracker” of the Centers for Disease Control and Prevention (CDC) [56]. Thus, the data reported here and our interpretation of a limited expansion potential of XBB.1.5 appear to be in keeping with the current epidemiological observations.

We also performed a structural analysis aimed to identify the main differences between XBB.1.5 and XBB/XBB.1. Overall, no evident difference could be detected between variants XBB.1 and XBB1.5 in the distribution of the electrostatic surface potential in NTD and RBD. Interestingly, however, the three XBB descendants differ from BA.2 for their negative net charge on NTD, in this case being similar to the first Omicron variant, the BA.1 [57]. A negative NTD electrostatic potential may indicate a decreased ability to interact with the negatively charged cellular components such as syalosides and the AXL receptor, as previously discussed [57]. The XBB.1 and XBB.1.5 RBDs differ at sequence position 486, which is occupied by Ser and Pro, respectively, whereas BA.2 displays the original Whuan residue Phe. The conserved F486 in BA.2 can establish van der Waals contacts with residues in a hydrophobic pocket on the ACE2 receptor. The substituting Ser in XBB and XBB.1 tend to destabilize the RBD–ACE2 interface by disrupting hydrophobic interactions. Disrupted hydrophobic interactions are in part restored by the Pro in XBB.1.5, although PRODIGY does not assign it to the interface. Consistently, the interaction BA.2 RBD–ACE2 is inferred to be the most stable among the four variants. The XBB.1.5 Pro486 may play the role of stiffening the loop in which it is contained, and it may be speculated that the partial increase in the stability induced by Pro may also have an entropic component. Indeed, the decrease in loop flexibility may decrease its loss of entropy upon complex formation. However, the effect on the binding interface is moderate as position 486 is not predicted to be a hotspot. Other authors [58] have reported a higher ACE2 binding affinity of XBB.1.5 compared with XBB and XBB,1, a finding that could keep in with our observation about the partial recovery of hydrophobic interactions by proline replacement of phenylalanine discussed above. However, our in-depth genomic and evolutionary analysis is not in keeping with the above authors’ forecast of XBB.1.5 being able to cause the next global wave of the pandemic [57].

Comparative immunoinformatics analyses suggest that no evident difference among the four variants for the B-epitopes and the MHC1 binding peptides can be detected. Only in one case did a weak MHC-1 binder present in BA.2 disappear in the other variants. While displaying a particularly strong evasion from neutralizing antibodies [22], XBB.1.5, as its predecessors’ variants, has almost entirely preserved the T cell epitopes, which play an important protective role against severe COVID-19 disease [22].

## 5. Conclusions

Nowadays, the Omicron variant of concern, particularly BA.5 and its descendant lineages, still appear to be the dominant variants circulating globally [1], and XBB.1.5 after a few months seems to spread even more slowly than other sub-variants that have caused concerns in 2022. Collectively, our genomic, genetic and structural analyses do not provide evidence for a particularly high risk of XBB.1.5 expansion to become a new, global, public health threat. However, such as for all circulating variants, it cannot be excluded that new further mutations will occur and make XBB.1.5 more dangerous. In such a context, the genome-based survey under an integrated multidisciplinary approach must continue uninterruptedly because it is the only way to identify new lineages and/or predict important changes in the viral genomic make-up of SARS-CoV-2.

## Figures and Tables

**Table 1:** Net charge of NTD and RBD. Comparison of the net charge of NTD and RBD for BA.2, XBB, XBB.1 and XBB.1.5.

	BA.2	XBB	XBB.1	XBB.1.5
NTD	0.95 ± 0.04	1.14 ± 0.04	1.18 ± 0.04	1.18 ± 0.04
RBD	5.19 ± 0.01	5.45 ± 0.02	5.45 ± 0.02	5.42 ± 0.01

**Table 2:** Predicted RBD–ACE2 interaction energy. Values of interaction energy are expressed in Kcal/mol.

	BA.2	XBB	XBB.1	XBB.1.5
Foldx 5.0	−6.19 ± 0.32	−3.54 ± 0.30	−3.54 ± 0.30	−4.57 ± 0.27
PRODIGY	−11.70 ± 0.05	−11.48 ± 0.05	−11.48 ± 0.05	−10.84 ± 0.05
MM/GBSA	−68.43 ± 2.10	−60.82 ± 0.99	−60.82 ± 0.99	−62.44 ± 2.28

**Table 3:** Contribution of position 486 to the RBD–ACE2 stability. Values are expressed in Kcal/mol.

	BA.2	XBB	XBB.1	XBB.1.5
MM/GBSA	−3.96 ± 0.01	−0.72 ± 0.20	−0.47 ± 0.12	−1.41 ± 0.11

## Data Availability

Genomes analyzed in the present study were taken from GSAID database and are available at https://gisaid.org/ (accessed on 13 January 2023).

## Supplementary Materials

The following supporting information can be downloaded at: https://www.mdpi.com/article/10.3390/microorganisms11040912/s1, Figure S1: Phylogenomic time-scaled reconstruction of XBB.1.5. Highlight of the GSAID Clade 23 A in a time-scaled phylogenetic tree of a representative global subsample of 141 SARS-CoV-2 XBB.1.5 genomes sampled between October 2022 and February 2023 labeled according to WHO regions of origin. The tree was edited and visualized using FigTree 1.4.0 [30]. The generated figure was edited using the software GIMP 2.8 (available at https://www.gimp.org/downloads/oldstable/, accessed on 20 February 2023). Figure S2: Projection on a two-dimensional map of the electrostatic potential surface of the NTDs of the four variants. Color scale is reported aside the BA.2 map. Electrostatic potential values are expressed as kT/e units. Map axes report the projected polar coordinates of the domains. Table S1: Spike variant mutations of compared lineages. File S1: Acknowledgement and reference of authors who deposited analyzed genomes in GISAID portal.
