# Supplementary material for: Molecular In-Depth on the Epidemiological Expansion of SARS-CoV-2 XBB.1.5
Source: Microorganisms. 2023 Mar 31;11(4):912. doi: 10.3390/microorganisms11040912 (PMC10142263; doi:10.3390/microorganisms11040912)
Supplement: Supplementary file 1 [file microorganisms-11-00912-s001.zip › File_S1.pdf]

SUPPLEMENTAL TABLE

Data Availability

GISAID Identifier:	EPI_SET_230113ms
doi:	10.55876/gis8.230113ms

All genome sequences and associated metadata in this dataset are published in GISAID's EpiCoV database. To view the contributors of each individual sequence with details such as accession number, Virus name, Collection date, Originating Lab and Submitting Lab and the list of Authors, visit 10.55876/gis8.230113ms

Data Snapshot

- EPI_SET_230113ms is composed of 50 individual genome sequences.
- The collection dates range from 2022-04-01 to 2022-11-30;
- Data were collected in 13 countries and territories;
- All sequences in this dataset are compared relative to hCoV-19/Wuhan/WIV04/2019 (WIV04), the official reference sequence employed by GISAID (EPI_ISL_402124). Learn more at https://gisaid.org/WIV04.

SUPPLEMENTAL TABLE

Data Availability

GISAID Identifier: EPI_SET_230113ch
doi: 10.55876/gis8.230113ch

All genome sequences and associated metadata in this dataset are published in GISAID's EpiCoV database. To view the contributors of each individual sequence with details such as accession number, Virus name, Collection date, Originating Lab and Submitting Lab and the list of Authors, visit 10.55876/gis8.230113ch

Data Snapshot

- EPI_SET_230113ch is composed of 50 individual genome sequences.
- The collection dates range from 2022-11-21 to 2022-12-31;
- Data were collected in 12 countries and territories;
- All sequences in this dataset are compared relative to hCoV-19/Wuhan/WIV04/2019 (WIV04), the official reference sequence employed by GISAID (EPI_ISL_402124). Learn more at https://gisaid.org/WIV04.

SUPPLEMENTAL TABLE

Data Availability

GISAID Identifier:	EPI_SET_230113rz
doi:	10.55876/gis8.230113rz

All genome sequences and associated metadata in this dataset are published in GISAID's EpiCoV database. To view the contributors of each individual sequence with details such as accession number, Virus name, Collection date, Originating Lab and Submitting Lab and the list of Authors, visit 10.55876/gis8.230113rz

Data Snapshot

- EPI_SET_230113rz is composed of 50 individual genome sequences.
- The collection dates range from 2022-10-16 to 2022-12-27;
- Data were collected in 15 countries and territories;
- All sequences in this dataset are compared relative to hCoV-19/Wuhan/WIV04/2019 (WIV04), the official reference sequence employed by GISAID (EPI_ISL_402124). Learn more at https://gisaid.org/WIV04.

SUPPLEMENTAL TABLE

Data Availability

GISAID Identifier: EPI_SET_230113nr
doi: 10.55876/gis8.230113nr

All genome sequences and associated metadata in this dataset are published in GISAID's EpiCoV database. To view the contributors of each individual sequence with details such as accession number, Virus name, Collection date, Originating Lab and Submitting Lab and the list of Authors, visit 10.55876/gis8.230113nr

Data Snapshot

- EPI_SET_230113nr is composed of 50 individual genome sequences.
- The collection dates range from 2022-08-16 to 2023-01-03;
- Data were collected in 14 countries and territories;
- All sequences in this dataset are compared relative to hCoV-19/Wuhan/WIV04/2019 (WIV04), the official reference sequence employed by GISAID (EPI_ISL_402124). Learn more at https://gisaid.org/WIV04.

SUPPLEMENTAL TABLE

Data Availability

GISAID Identifier:	EPI_SET_230113qr
doi:	10.55876/gis8.230113qr

All genome sequences and associated metadata in this dataset are published in GISAID's EpiCoV database. To view the contributors of each individual sequence with details such as accession number, Virus name, Collection date, Originating Lab and Submitting Lab and the list of Authors, visit 10.55876/gis8.230113qr

Data Snapshot

- EPI_SET_230113qr is composed of 50 individual genome sequences.
- The collection dates range from 2022-11-05 to 2023-01-05;
- Data were collected in 12 countries and territories;
- All sequences in this dataset are compared relative to hCoV-19/Wuhan/WIV04/2019 (WIV04), the official reference sequence employed by GISAID (EPI_ISL_402124). Learn more at https://gisaid.org/WIV04.

SUPPLEMENTAL TABLE

Data Availability

GISAID Identifier:	EPI_SET_230113nv
doi:	10.55876/gis8.230113nv

All genome sequences and associated metadata in this dataset are published in GISAID's EpiCoV database. To view the contributors of each individual sequence with details such as accession number, Virus name, Collection date, Originating Lab and Submitting Lab and the list of Authors, visit 10.55876/gis8.230113nv

Data Snapshot

- EPI_SET_230113nv is composed of 288 individual genome sequences.
- The collection dates range from 2022-08-27 to 2023-01-02;
- Data were collected in 23 countries and territories;
- All sequences in this dataset are compared relative to hCoV-19/Wuhan/WIV04/2019 (WIV04), the official reference sequence employed by GISAID (EPI_ISL_402124). Learn more at https://gisaid.org/WIV04.

SUPPLEMENTAL TABLE

Data Availability

GISAID Identifier: EPI_SET_230113vm
doi: 10.55876/gis8.230113vm

All genome sequences and associated metadata in this dataset are published in GISAID's EpiCoV database. To view the contributors of each individual sequence with details such as accession number, Virus name, Collection date, Originating Lab and Submitting Lab and the list of Authors, visit 10.55876/gis8.230113vm

Data Snapshot

- EPI_SET_230113vm is composed of 50 individual genome sequences.
- The collection dates range from 2022-11-30 to 2023-01-06;
- Data were collected in 15 countries and territories;
- All sequences in this dataset are compared relative to hCoV-19/Wuhan/WIV04/2019 (WIV04), the official reference sequence employed by GISAID (EPI_ISL_402124). Learn more at https://gisaid.org/WIV04.

SUPPLEMENTAL TABLE

Data Availability

GISAID Identifier:	EPI_SET_230113ef
doi:	10.55876/gis8.230113ef

All genome sequences and associated metadata in this dataset are published in GISAID's EpiCoV database. To view the contributors of each individual sequence with details such as accession number, Virus name, Collection date, Originating Lab and Submitting Lab and the list of Authors, visit 10.55876/gis8.230113ef

Data Snapshot

- EPI_SET_230113ef is composed of 50 individual genome sequences.
- The collection dates range from 2022-09-08 to 2022-11-30;
- Data were collected in 16 countries and territories;
- All sequences in this dataset are compared relative to hCoV-19/Wuhan/WIV04/2019 (WIV04), the official reference sequence employed by GISAID (EPI_ISL_402124). Learn more at https://gisaid.org/WIV04.

SUPPLEMENTAL TABLE

Data Availability

GISAID Identifier:	EPI_SET_230113da
doi:	10.55876/gis8.230113da

All genome sequences and associated metadata in this dataset are published in GISAID's EpiCoV database. To view the contributors of each individual sequence with details such as accession number, Virus name, Collection date, Originating Lab and Submitting Lab and the list of Authors, visit 10.55876/gis8.230113da

Data Snapshot

- EPI_SET_230113da is composed of 156 individual genome sequences.
- The collection dates range from 2022-08-24 to 2023-01-07;
- Data were collected in 12 countries and territories;
- All sequences in this dataset are compared relative to hCoV-19/Wuhan/WIV04/2019 (WIV04), the official reference sequence employed by GISAID (EPI_ISL_402124). Learn more at https://gisaid.org/WIV04.

SUPPLEMENTAL TABLE

Data Availability

GISAID Identifier:	EPI_SET_230113re
doi:	10.55876/gis8.230113re

All genome sequences and associated metadata in this dataset are published in GISAID's EpiCoV database. To view the contributors of each individual sequence with details such as accession number, Virus name, Collection date, Originating Lab and Submitting Lab and the list of Authors, visit 10.55876/gis8.230113re

Data Snapshot

- EPI_SET_230113re is composed of 208 individual genome sequences.
- The collection dates range from 2022-11-08 to 2023-01-09;
- Data were collected in 7 countries and territories;
- All sequences in this dataset are compared relative to hCoV-19/Wuhan/WIV04/2019 (WIV04), the official reference sequence employed by GISAID (EPI_ISL_402124). Learn more at https://gisaid.org/WIV04.

SUPPLEMENTAL TABLE

Data Availability

GISAID Identifier:	EPI_SET_230113yu
doi:	10.55876/gis8.230113yu

All genome sequences and associated metadata in this dataset are published in GISAID's EpiCoV database. To view the contributors of each individual sequence with details such as accession number, Virus name, Collection date, Originating Lab and Submitting Lab and the list of Authors, visit 10.55876/gis8.230113yu

Data Snapshot

- EPI_SET_230113yu is composed of 8 individual genome sequences.
- The collection dates range from 2023-01-07 to 2023-01-08;
- Data were collected in 3 countries and territories;
- All sequences in this dataset are compared relative to hCoV-19/Wuhan/WIV04/2019 (WIV04), the official reference sequence employed by GISAID (EPI_ISL_402124). Learn more at https://gisaid.org/WIV04.
