# Supplementary material for: Molecular In-Depth on the Epidemiological Expansion of SARS-CoV-2 XBB.1.5
Source: Microorganisms. 2023 Mar 31;11(4):912. doi: 10.3390/microorganisms11040912 (PMC10142263; doi:10.3390/microorganisms11040912)
Supplement: Supplementary file 1 [file microorganisms-11-00912-s001.zip › Table_S1.pdf]

Table S1. Spike variant mutations of compared lineages.

	BA.2	XBB	XBB.1	XBB.1.5
**NTD**	T19I	T19I	T19I	T19I
	L24S	L24S	L24S	L24S
	P25	P25	P25	P25
	P26	P26	P26	P26
	A27	A27	A27	A27
		V83A	V83A	V83A
	G142D	G142D	G142D	G142D
		Y144	Y144	Y144
		H146Q	H146Q	H146Q
		Q183E	Q183E	Q183E
	V213G	V213E	V213E	V213E
			G252V	G252V
**RBD**	G339D	G339H	G339H	G339H
		R346T	R346T	R346T
		L368I	L368I	L368I
	S371F	S371F	S371F	S371F
	S373P	S373P	S373P	S373P
	S375F	S375F	S375F	S375F
	T376A	T376A	T376A	T376A
	D405N	D405N	D405N	D405N
	R408S	R408S	R408S	R408S
	K417N	K417N	K417N	K417N
	N440K	N440K	N440K	N440K
		V445P	V445P	V445P
		G446S	G446S	G446S
		N460K	N460K	N460K
	S477N	S477N	S477N	S477N
	T478K	T478K	T478K	T478K
	E484A	E484A	E484A	E484A
		F486S	F486S	**F486P**

		F490S	F490S	F490S
	Q493R			
	Q498R	Q498R	Q498R	Q498R
	N501Y	N501Y	N501Y	N501Y
	Y505H	Y505H	Y505H	Y505H
	D614G	D614G	D614G	D614G
	H655Y	H655Y	H655Y	H655Y
	N679K	N679K	N679K	N679K
	P681H	P681H	P681H	P681H
	N764K	N764K	N764K	N764K
	D796Y	D796Y	D796Y	D796Y
	Q954H	Q954H	Q954H	Q954H
	N969K	N969K	N969K	N969K
