# Supplementary figures and images for: Molecular In-Depth on the Epidemiological Expansion of SARS-CoV-2 XBB.1.5
Source: Microorganisms. 2023 Mar 31;11(4):912. doi: 10.3390/microorganisms11040912 (PMC10142263; doi:10.3390/microorganisms11040912)

# Phylogenomics of SARS-CoV-2 XBB.1.5 (GSAID CLADE 23A)

## WHO Regions

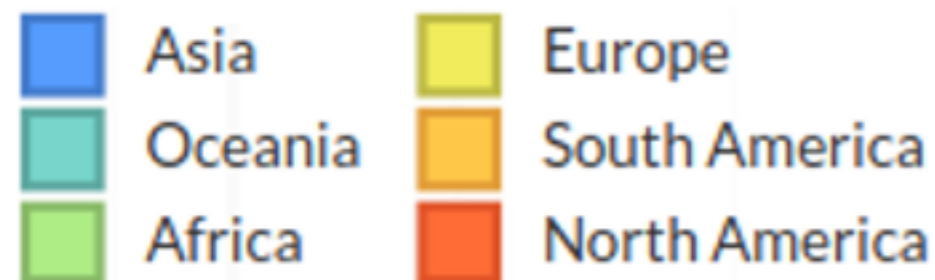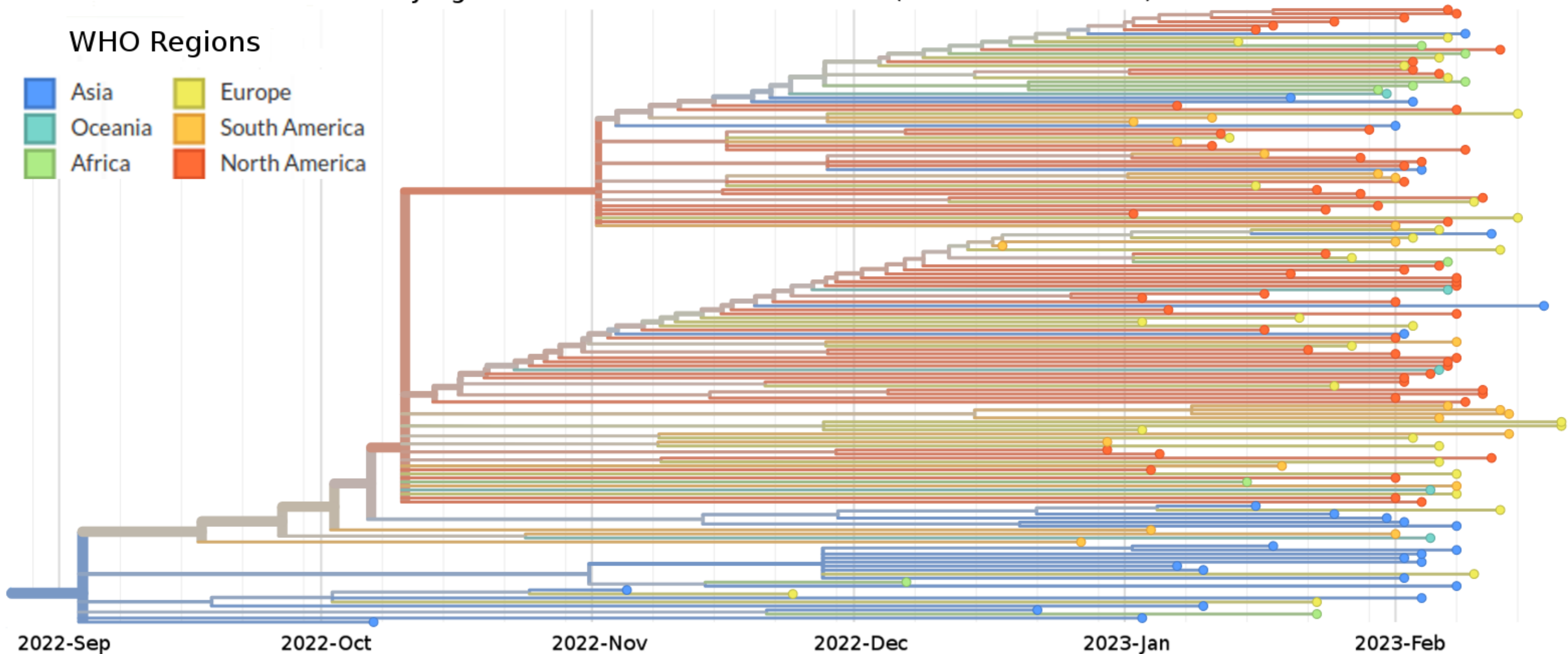

Supplement: Supplementary file 1 [file microorganisms-11-00912-s001.zip › Figure_S1.pdf]

**BA.2**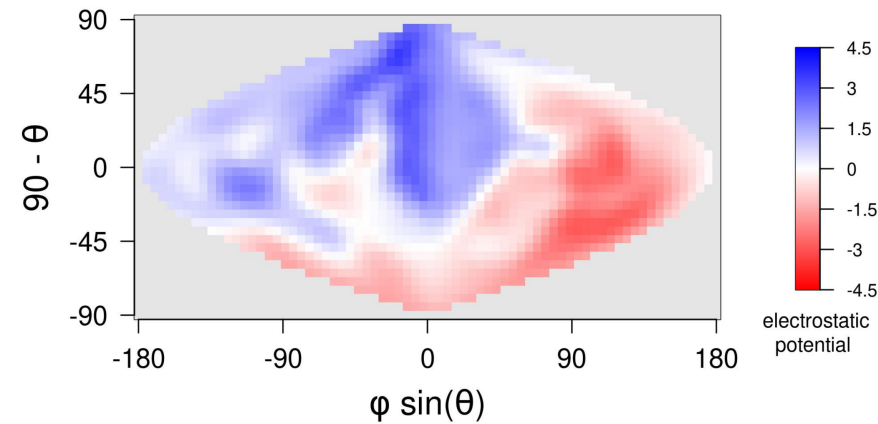**XBB**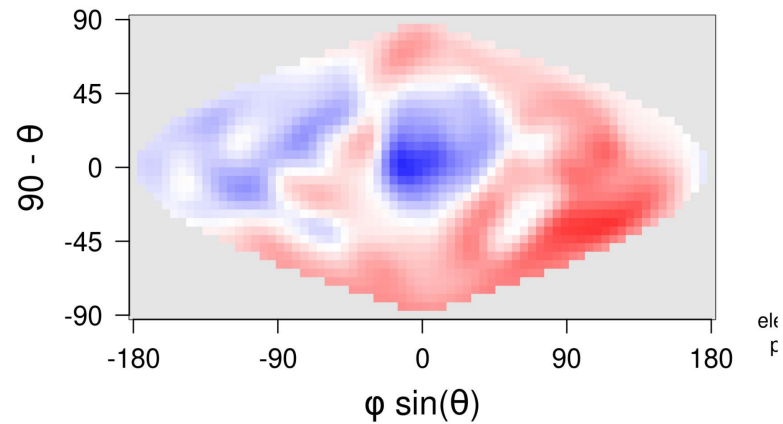**XBB.1/XBB.1.5**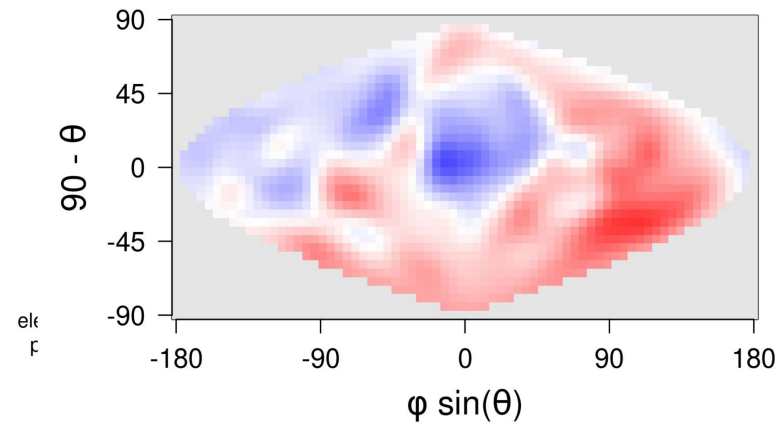

Supplement: Supplementary file 1 [file microorganisms-11-00912-s001.zip › Figure_S2.pdf]
